# Advances in Photonic and Plasmonic Nanomaterials

**DOI:** 10.3390/nano15010055

**Published:** 2024-12-31

**Authors:** Nikolaos G. Semaltianos

**Affiliations:** Department of Physics, Aristotle University of Thessaloniki, 54124 Thessaloniki, Greece; n.semaltianos@email.com

Nanomaterials and nanostructures with different morphologies or geometrical shapes that exploit plasmons, and particularly surface plasmon polaritons, are useful for a wide range device fabrication-related applications. They have been intensively investigated because they provide unique optical and photonic properties, such as largely amplified local electric fields near a metal–dielectric interface, efficient control of light guiding into nanoscale volumes, ultrasensitivity of light to external parameters, easymanipulation of optical characteristics via structure geometrical parameters, and others. Furthermore, photonic nanomaterials can exhibit size-tunable light emission and unique non-linear optical properties. Combining photonic and plasmonic nanomaterials results in their emission enhancement.

This Special Issue collected articles that investigated novel photonic and plasmonic nanomaterials and nanostructures with advanced properties for better performance. Ten articles were published in Volume I and six in Volume II. 

Specifically, the articles published in this Special Issue dealt with the fabrication of excitonic molecule-coated plasmonic nanomaterials [1], quantum dots in nanowires coupled with plasmonic nanocavities [2], linear tunability of Fano resonances [3], plasmonic nanocavities coupled to metal–insulator–metal structures [4], plasmons in graphene-covered nanowire pairs with a substrate [5], vortex beam-encoded all-optical logic gates based on plasmonic nanostructures [6], infrared absorbers based on reactive impedance surfaces [7], optical ring resonators in high thermo-optic coefficient materials [8], semiconductor plasmonic nanowire lasers with end-facet mirrors [9], the utilization of black phosphorus instead of graphene in Fano resonance-based devices [10], the non-linear optical response of bi-dimensional layered transition metal dichalcogenides [11], the optical properties of double-line waveguides inside rare-earth ion-doped glass with embedded Ag nanoparticles [12] or Ag nanoclusters [13], random lasing from rare-earth ion-doped glass [14], the characterization of the electrical, structural, and optical properties of indium tin oxide thin films [15], and the extraordinary optical transmission through plasmonic nanostructures [16]. A brief summary of each article published in this Special Issue, including recent developments in the field, the gaps in knowledge, and how the article addresses those gaps, with a primary focus on future research areas, is provided below.

Quantum networks or other devices used in quantum technologies for quantum information processing usually utilize bare plasmonic nanomaterials as building blocks. In this Special Issue, ref. [1] proposed and experimentally demonstrated the fabrication of a simple plasmon–exciton coupling system consisting of J-aggregate excitonic molecule-coated (layer thickness ~4 nm) plasmonic Au nanocubes (average side length of ~85 nm) (AuNC@J-agg) on a Au nanofilm (~100 nm) (AuNC@J-agg/AuNF) that can be used as a building block for the construction of more complex quantum technology systems. The AuNC@J-agg/AuNF system exhibits a ~2.5 times stronger plasmon–exciton coupling strength (Rabi splitting of 345–377 meV) than the AuNC@J-agg system (Rabi splitting of 100–140 meV).

Quantum dots as single-photon emitters embedded in nanowires (QDNWs) allow for their positional and dimensional controllability in photonic nanostructures. The Purcell effect in the framework of cavity quantum electrodynamics (QED) indicates that the spontaneous emission rate of a single-photon emitter can be enhanced by placing it in an optical resonant metal nanocavity. The authors of [2] used an In_0.3_Ga_0.7_As quantum dot (diameter = 40 nm; thickness = 8 nm) embedded in the middle of a GaAs nanowire (diameter = 70 nm) as the single-photon emitter and placed it in a wire-groove nanocavity (dimensions below the free-space wavelength: width = 10 nm; thickness = 40 nm (equal to the diameter of the quantum dot); length = 259 nm) carved on a Ag film on a silica substrate perpendicular to the nanowire at the quantum dot site to enhance its emission rate. Theoretical analysis showed a large enhancement of the emission rate—by 617 times—induced by the nanocavity, which resulted in an optimized maximum emission rate of 50.2 GHz. This makes the structure suitable for utilization in quantum information systems and networks working at the speed of tens of gigabits per second.

Typically, plasmon resonance in nanoscale devices based on surface plasmon polaritons (SPPs) is of a symmetric and relatively wide quasi-Lorentzian type, which limits the applications of these devices. Fano-type resonance modes are instead characterized by a sharp and asymmetric line shape, which also exhibits a strong sensitivity to background material changes, thus making devices based on this type of resonance more useful in terms of their applications. However, even in structures that are based on Fano resonance, tunability has either been neglected so far or is difficult to achieve in practice. Furthermore, the linear tunability of plasmon resonances is the simplest to realize in devices, since it can be achieved by using a linear control voltage without requiring a non-linear scale and thus has important applications. Thus, in [3], the authors proposed and numerically analyzed an ultracompact plasmonic nanostructure (Ag) to obtain multiple linearly tunable Fano resonance modes using transfer matrix theory and finite element method (FEM) simulations. The nanostructure consists of a waveguide loaded with two rectangular cavities coupled by a circular cavity with a metal-strip core in a metal–insulator–metal (MIM) structure, which provides four Fano resonances between 585 and 761 nm originating from the coupling effect of the narrow modes in the metal-core circular cavity and the wide modes in the rectangular cavities. The Fano resonances exhibit a dependence on the cavity’s geometrical parameters, and their line profile can be linearly tuned by adjusting the orientation angle of the metal core in the circular cavity, while the figure of merit (FOM) exhibits a linear increase with the orientation angle, with a maximum value of 8056. The proposed plasmonic system has high transmission, is ultracompact and easily integrable with other devices, and can find applications in biochemical detection or sensing, ultra-fast switching, modulators, and slow-light technologies.

Plasmonic nanostructures that exhibit multiple resonance modes have important applications since they can provide devices with multi-frequency operation and greater flexibility. Usually, the traditional method to obtain plasmonic multimodes involves combining several identical or different resonance elements such as waveguides, nanodisk cavities, or rectangular shape resonators with appropriately varying dimensions or characteristics. This strategy has the disadvantage of a difficulty in appropriately tuning the optical properties of the elements, and it also results in a relatively large structure size, imposed by the need to use many elements to increase the number of modes, thus limiting device integrability. Therefore, using FEM simulations, the authors of [4] proposed and numerically analyzed a type of plasmonic nanostructure (Ag) consisting of a completely different type of resonance elements that exhibits multiple resonance modes closely distributed in a broad spectral range. It consists of a fan-shaped cavity coupled to a MIM waveguide. As many as ten modes with wavelengths from 502 to 785 nm and an average interval of about 30 nm were obtained, originating from the coexistence and interference of three types of basic modes in the structure: the ring waveguide modes, the modes in a ring array of periodic metal–air grooves, and the metal-core circular cavity modes. The authors characterized in detail the dependence of the resonance modes on the geometric parameters of the structure. An effective modulation of the resonance modes was achieved by adjusting the axial offset of the metal core in the circular, and thus fan-shaped, cavity. Applications include optical filters, ultrafast switches, biochemical sensors, and data storage.

Contrary to the noble metals that are usually used in plasmonic components, which suffer from large Ohmic losses and a lack of tunability, graphene plasmons exhibit remarkable properties such as extremely strong mode confinement, significant field enhancement, and tunability. Particularly, nanomaterials consisting of graphene-covered nanowires (GCNWs) which are also combined with a buffer or substrate are characterized by a large propagation length modes and an ultrasmall size, which becomes even smaller when nanowire pairs (dimers) are used. However, so far, studies of GCNW pairs with a substrate have been very limited. Thus, the authors of [5] theoretically investigated and demonstrated a high-performance and low-loss transmission of graphene plasmons, which they achieved by adding a silica substrate to a GCNW pair (radius = 50–150 nm). The losses associated with the substrate were compensated for by the insertion of a low-index material layer (e.g., air) between the nanowire and substrate. The authors systematically investigated the dependence of the mode properties on substrate thickness, gap size, nanowire radius, frequency, and chemical potential using FEM simulations. A propagation length of 15 µm and an ultrasmall normalized mode area of ~10^−4^ could be obtained at 30 THz. Such plasmonic nanostructures can find applications in tunable integrated nanophotonic devices.

Vortex beam-encoded all-optical logic gates are very important for future information processing. Currently, only a few logic devices are encoded by vortex beams. Additionally, bulky optical elements such as mirrors, dove prisms, and pentaprisms are used in these devices, and they are not fully compatible with device integration technology. The authors of [6] proposed an integrated vortex beam-encoded all-optical logic gate based on a nano-ring plasmonic antenna, which has the same circular geometry as the intensity distribution of the vortex beam. By defining the two circular polarization states of the input vortex beam as the input logic states and the normalized intensity of the plasmonic field at the center of the nano-ring as the output logic states, OR and AND (NOR and NAND) logic gates were obtained when two first-order vortex beams werechosen as the input signals. A NOT logic gate was obtained when one first-order vortex beam was chosen as the input signal. Additionally, by defining the two linear polarization states (*x* and *y* polarization) of the input vortex beams as the two input logic states, an XNOR logic gate was achieved when two first-order vortex beams were chosen as the two input signals.

Infrared (IR) absorbers based on MIM have been widely investigated due to their simplicity and relatively high efficiency. However, those based on ultrathin spacers (to achieve perfect absorption) suffer from low field enhancement. Thus, in [7], the authors proposed a new MIM absorber structure that utilizes a reactive impedance surface (RIS) to achieve high electric field enhancement and near-perfect absorption. The unit cell consists of a RIS, which is an array (8 × 8) of gold metal squares (dimensions of 60 × 60 nm; periodicity = 125 nm) located at the middle of a grounded SiO_2_ slab (thickness of 50 and 50 nm above and below the metal squares, respectively; dimensions of 1 × 1-μm) on top of a reflector gold substrate (thickness = 200 nm; same dimensions), and a gold nanodisk (diameter = 235 nm; thickness = 10 nm) located on top of the dielectric slab at its center. With a filling factor of 4.33%, the structure provides an electric field enhancement factor of 180 with an absorption of 98% at 230 THz and is polarization-independent. These plasmonic nanostructures find applications in sensors and surface-enhanced IR spectroscopy.

Optical (micro)ring resonators (ORRs), also made of high-thermo-optic-coefficient materials, can be used as highly sensitive temperature-sensing devices because they exhibit an enhanced light–matter interaction due to the strong electromagnetic field in resonators with a high optical quality factor (Q-factor). This implies that an accurate modeling of their temperature sensitivity in particular is of primary importance before fabrication. In [8], the authors numerically investigated the temperature sensitivity of ORRs made of Si, diamond, or GaN, as well as studying the influence of self-heating due to two-photon absorption (TPA). They used different FEMs, which require low computational resources and provide fast results, to calculate the free spectral range (FSR) of the resonator, the number of longitudinal modes (*m*), and the shift of the resonance wavelength (1550 nm) due to the change in temperature. Due to its high thermo-optic coefficient, Si exhibits a higher temperature sensitivity (68.2 pm·K^−1^) compared to diamond (16.8 pm·K^−1^) and GaN (30.4 pm·K^−1^), but in diamond or GaN, self-heating at a resonance wavelength is less critical above a threshold intensity due to the absence of an intrinsic TPA. Furthermore, due to its high thermal conductivity, low thermal expansion coefficient, and wide band gap, diamond can tolerate high optical power at resonance while achieving a high Q-factor.

Plasmonic nanowire (NW) lasers on a dielectric nano-gap on a metallic substrate are important nanoscale devices, but investigations of their end-facet reflectivity in particular, which usually exhibits a fast decrease with the decrease in NW diameter, are still very limited. Thus, using 3D finite-difference time-domain (FDTD) simulations, the authors of [9] proposed and studied a miniaturized hybrid plasmonic GaAs/AlGaAs core–shell NW laser with high-reflectivity gold cavity end-mirrors on a Ag substrate separated with a nanoscale MgF_2_ dielectric gap layer. Due to photon–plasmon coupling, the electric field intensity in the dielectric gap layer increases, resulting in a much lower threshold gain, while the extraction efficiency remains high, especially for small diameters, since the mode energy is also mainly concentrated in the gap. For diameters in the range of 100–150 nm, the threshold gain is reduced by 60–80%, whereas the output power is maintained at >60% compared to that in NW without end-mirrors. Such microscale devices find applications in ultracompact photonic integrated circuits.

Fano resonance, with its sharp asymmetric line shape, high sensitivity, and large FOM, also finds important applications in high-performance biochemical THz sensors. Although 2D materials exhibit much stronger plasmon confinement than metals, the low efficiency of light-excited plasmons in graphene limits its applicability in Fano resonance-based devices compared to black phosphorous (BP), which is characterized by a high coupling efficiency and low scattering losses. In addition, black phosphorous is particularly attractive for frequency-tunable nanostructures because its Fermi level can be changed by chemical doping to further modify its photoelectric properties. Thus, in [10], the authors used FDTD simulations to investigate a proposed THz sensor based on Fano resonance caused by coupling the dipole plasmon bright mode of an annular Au ring with the quadrupole plasmon dark mode of a bowtie BP monolayer. The amplitude and frequency of the resonance were precisely adjusted by relating the geometrical characteristics of the Au ring (inner and outer diameter) to those of BP (side length) or its Fermi level, determined by the electron doping concentration, which caused a change in its refractive index. A FOM of 69.3 and a maximum sensitivity of 9.3 μm/refractive index unit (RIU) were achieved.

The non-linear optical (NLO) response of nanomaterials leads to a variety of effects that form the basis for the operation of many optical components. Bi-dimensional layered transition metal dichalcogenides (2D-LTMDs) have most recently appeared as beyond-graphene nanomaterials with semiconducting and metallic optical properties. In [11], the authors mainly reviewed their own work investigating the NLO response of a series of 2D-LTMDs (MoS_2_, MoSe_2_, MoTe_2_, WS_2_, semimetallic WTe_2_, ZrTe_2_, and metallic NbS_2_ and NbSe_2_) in suspension using different NLO techniques of Fourier transform non-linear optics, Rayleigh scattering, Z-scan, photoacoustic Z-scan, optical Kerr gate, and spatial self-phase modulation. The NLO response from a thermal to non-thermal origin was studied, and the non-linear refraction index and absorption coefficient were measured and analyzed.

Double-line waveguides, consisting of pairs of lines—each of which is the result of four or eight laser beam overscans written inside Nd^+3^-doped GeO_2_-PbO glass by a scanning femtosecond (fs) (800 nm/30 fs/10 kHz/30 μJ/0.5 mm·s^−1^) laser beam—exhibit positive internal gains. Thus, these structures are promising for applications in photonics and optoelectronics. Furthermore, rare-earth ion-doped GeO_2_-based glasses doped with Ag nanoparticles (NPs) generally exhibit photoluminescence (PL) enhancement due to the plasmonic effect of NPs. Thus, the authors of [12] investigated the influence of double-line waveguides written inside a Nd^+3^-doped GeO_2_-PbO glass, but also with embedded Ag NPs, on their optical properties. At 1064 nm (808 nm pump laser, 600 mW), the PL was enhanced by ~47% and gain by ~40% and ~30% in waveguides with four and eight superimposed lines, respectively, compared with those in glass without NPs. Structures with embedded plasmonic NPs are better for photonic applications, particularly for the manufacturing of lasers and integrated amplifiers.

Ag nanoclusters are nanomaterials consisting of a few tens of Ag atoms and Ag^+^ free ions. They exhibit a broad-band PL emission that covers the visible spectral range and is not only size- but also laser-excitation-wavelength-dependent. Rare-earth ion-doped glasses with embedded Ag nanoclusters exhibit an amplified ion PL emission, attributed to the mechanism of energy transfer (ET) from the nanoclusters to the ions, and have important photonic applications. Composite structures consisting of a variety of glasses have been investigated so far, but GeO_2_-PbO glasses have unique and important optical characteristics. Thus, the authors of [13] investigated an Yb^+3^ ion-doped GeO_2_-PbO glass with embedded Ag nanoclusters. The tunability of the PL emission from blue to orange was demonstrated to depend on the concentration of Yb^+3^ ions due to its influence on the size of the Ag nanoclusters, which in turn affects the efficiency of ET from these to the Yb^+3^ ions: a higher Yb^+3^ ion concentration results in smaller Ag nanoclusters, whose excitation at 355 nm leads to more efficient ET to Yb^+3^ ions compared to excitation at 410 nm. Under 355 nm excitation, the sample with the highest Yb^+3^ ion concentration exhibited the highest PL intensity, at 980 nm, associated with Yb^+3^ ions, whereas under 410 nm excitation, the sample with the lowest Yb^−3^ ion concentration exhibited the highest PL intensity. These kinds of composite glasses have promising applications in tunable CW laser sources and photovoltaic devices.

Random lasers (RLs) find a wide range of applications in photonics and optoelectronics, mainly because they are easy and cheap to fabricate. Although high-efficiency RLs have been demonstrated in Nd-doped materials, the existing literature lacks investigations of RLs using glass. Glass presents several advantages: it is an inexpensive material compared to crystals, can be highly doped with rare-earth ions, and has high mechanical strength and suitable optical properties. However, it does involve the challenge of laser emission verification, a problem that can in turn be solved by investigating the lasing temporal behavior. Furthermore, Nd-doped materials usually exhibit a stimulated emission at 1064 nm, which occurs simultaneously with that at 1.3 μm. The authors of [14] demonstrated an RL made from a Nd^+3^-doped TeO_2_-ZnO-Al_2_O_3_ (TZA) glass powder emitting at 1337 nm (under 585 nm pumping) with a 5 ns pulse width and a lasing threshold of 0.81 ± 0.10 mJ/mm^2^ for 16 wt% Nd_2_O_3_ concentration. For high pump powers, the 1064 nm emission exhibited fast decay before reaching the lasing threshold. A laser maximum peak power of ~0.25 mW was obtained for a pumping fluence of ~3.4 mJ/mm^2^. The absence of 1064 nm emission was attributed to the strong sample heating by the pump laser, which resulted in the population of the lower energy level associated with the transition corresponding to this wavelength, hindering population inversion thus stimulated emission while favoring laser emission at 1337 nm.

Indium tin oxide (ITO) has recently attracted an interest as a photonic material. Its properties are largely dependent on its deposition method and conditions, but reports in the literature lack a connection of the optical, electrical, and electronic parameters of the films—such as carrier mobility, high-frequency permittivity, electron effective mass, collision frequency, and others—with deposition conditions. In [15], the authors presented a comprehensive study of the electrical, structural, and optical properties of ITO thin films deposited on glass substrates by DC magnetron sputtering in relation to deposition conditions by fabricating guided mode resonance grating structures on the films and analyzing them. The study provides a highly needed reference for the research community and opens an avenue for future studies on novel photonic applications of ITO, as well as of other transparent conducting oxides (TCOs).

Optical transmission with an efficiency improved by several orders of magnitude compared to that predicted by classical diffraction theory through different sub-wavelength nanostructures in metal films—termed extraordinary optical transmission (EOT)—is very interesting for many applications in photonics and optoelectronics. Furthermore, fs laser beams have proven very effective in manipulating the dynamic properties of surface plasmon resonances. The spatio-temporal dynamics of EOT using an fs laser dual-beam has been investigated on different nanostructures so far, but controlling it through the manipulation of a pulse-to-pulse correlation of the beam remains a challenge. Thus, the authors of [16] investigated this question theoretically in a two-slit plasmonic antenna milled on a Au thin film on silica substrate. Their study showed that, for a certain angle of incidence of the beam on the antenna, the EOT can be tunably controlled by manipulating the phase correlation of the crossed fs laser dual-beam through the two slits via the interference between the beam and the transiently excited SPP waves.

I would like to acknowledge all authors who contributed their work to this Special Issue, the reviewers who devoted their time to improving the manuscripts, and the Editorial Board of *Nanomaterials* for their support.
